# Enhancing Intracytoplasmic Sperm Injection Outcomes With Zeta Sperm Selection: A Case Report

**DOI:** 10.7759/cureus.64809

**Published:** 2024-07-18

**Authors:** Sudheer Singh, Nancy Nair, Charu Pareek, Akash More, Avanti Kalbande

**Affiliations:** 1 Clinical Embryology, Datta Meghe Institute of Higher Education & Research, Wardha, IND; 2 Clinical Embryology, Datta Meghe Institute of Higher Education and Research, Wardha, IND; 3 Obstetrics and Gynaecology, Shalini Tai Meghe Hospital and Research Centre, Nagpur, IND

**Keywords:** intracytoplasmic sperm injection, zeta potential, sperm sorting technique, embryo transfer, in vitro fertilization, assisted reproductive techniques

## Abstract

Sperm morphology significantly influences the fertilization capacity of male germ cells. Morphological abnormalities are frequently associated with an overproduction of reactive oxygen species (ROS), leading to further sperm damage and subsequent infertility. This case study examines a couple facing infertility, with male factor infertility identified as the primary issue, characterized by teratozoospermia and a high DNA fragmentation index (DFI). The objective was to assess the efficacy of zeta potential (ZP) as a sperm sorting technique for intracytoplasmic sperm injection (ICSI) in patients showing high DNA fragmentation. A 34-year-old male with abnormal sperm parameters underwent ICSI using the ZP technique for sperm separation, while his 28-year-old female partner received ovarian stimulation. This intervention resulted in the development of two good-quality blastocysts, resulting in a successful embryo transfer (ET) and a positive pregnancy outcome. Previous attempts using conventional assisted reproductive technologies (ART), including in vitro fertilization (IVF), followed by ICSI and ET, as well as other sperm selection methods, were not successful. The ZP-based approach demonstrated significant benefits by selecting spermatozoa with optimal parameters, such as negative membrane potential, thereby enhancing the success rate. This case emphasizes the advantages of personalized treatment strategies in managing male infertility and highlights the potential of advanced sperm sorting techniques in improving fertility outcomes.

## Introduction

Infertility is defined as the inability of a couple to conceive after one year of regular, unprotected sexual intercourse. Approximately 50% are significantly caused by male infertility factors [1]. Several causes of male infertility include aging, congenital anatomical defects, gonadotoxic exposures, endocrinopathies, testicular dysfunction, and lifestyle choices like alcohol and smoking [2-3]. A comprehensive evaluation of male infertility involves a complete medical history, a physical examination, and several laboratory tests, including a fructose test, a hormone profile, and semen analysis [4]. A semen analysis is part of the first-line assessment of male fertility [5]. The criteria for evaluating sperm quality include various factors such as volume, morphology, concentration, progressive motility, total motility, vitality, and sperm count [6]. The DNA fragmentation index (DFI) of sperm reflects the integrity of the genetic material and identifies possible damage to the sperm [7]. Sperm chromatin fragmentation is linked to reduced reproductive capacity in infertile men when compared to fertile men [8].

Teratozoospermia is the term for the portion of spermatozoa with a normal shape below the lower reference level of >4% [9]. Selecting high-quality sperm is one of the most essential elements of a successful assisted reproductive technology (ART) process since it represents a diverse group that includes a wide variety of defective sperm morphologies that impact the head, neck, and tail, either separately or with each other [10]. Mature sperm cells needed for intracytoplasmic sperm injection (ICSI) are identified by analyzing their motility and morphology in the given sperm samples. Other techniques have also been developed to assess sperm quality, such as evaluating the zeta potential (ZP), which measures the surface charge on sperm cells [11].

Spermatozoa with an increased negative membrane potential are fully developed and unharmed. We have linked spermatozoa ZP measurement with sperm quality examination using conventional methods, utilizing a Zetasizer Nano Malvern Zetasizer Pro analyzer (Malvern Panalytical, Malvern United Kingdom). Cell surface charge has been calculated using measures of electrophoretic mobility, or ZP. When an electric field is applied, cells move. This movement is visible and is connected to the surface charge of the cell. The ZP technique can improve both embryonic growth and the quality of sperm DNA integrity [12].

## Case presentation

Patient information

A couple, a 34-year-old male and a 28-year-old female, visited the Wardha Test Tube Baby Centre (WTTBC) in Maharashtra, India. They had been married for five years and have been facing secondary infertility, as they have been unsuccessful in conceiving despite not using any contraception for the past three years. Their primary concern was seeking effective infertility treatment. They received a detailed description of all available ART treatments, including their advantages and disadvantages, and informed consent was obtained.

Medical history and timeline

The female had a history of ectopic pregnancy. The couple was trying to conceive naturally by 2019; they had undergone intrauterine inseminations (IUIs) and in vitro fertilization (IVF) procedures in 2021. They had two unsuccessful IUIs and one unsuccessful IVF attempt, despite all these years of ongoing treatments. The female partner's menstrual cycle was regular at ±28 days, and they had a previous history of partial salpingectomy. There was no family history of genetic abnormalities or medical issues in either of the individuals. There was no history of sexually transmitted infections in both partners. The male patient occasionally smoked and consumed alcohol, and the female partner had a healthy lifestyle. A male patient was diagnosed with abnormal sperm parameters based on a semen examination. In November 2022, the patient visited our clinic for further treatment. A diagnostic assessment was performed on both partners to evaluate their treatment strategies.

Diagnostic assessment

The male patient was advised to undergo a semen analysis and blood test to assess semen parameters and hormonal profile to investigate the underlying cause of infertility. The semen analysis result showed a total sperm count of 39 million/milliliters (mil/mL), with 75% total motility, and 97% of sperm morphology was found to be teratozoospermia, indicating abnormal sperm parameters according to WHO guidelines 2021 [13]. The semen analysis report is shown in Table 1. The hormonal profile of the male patient revealed that all hormonal levels were within normal ranges, including testosterone, and no signs of hypogonadism were observed.

**Table 1:** Semen analysis parameters pH: potential of hydrogen; ml: milliliter; PR: progressive motility; NP: non-progressive motility

Parameter	Findings	Reference limit
Volume	1.2ml	≥1.5 ml
pH	7.5	7.2-7.8
Liquefication	30 minutes	<30 minutes
Total sperm count	39 million	>15 million/ml
Total motility (PR+NP)	50%	>42%
Progressive motility	33%	>30%
Morphology	3%	>4%
DFI value	35%	<30%

The female patient's hormonal profile indicated the following levels: luteinizing hormone (LH) was 4.2 mIU/mL, testosterone was 22 ng/dL, progesterone was 0.4 ng/mL, estradiol was 110 pg/mL, follicle-stimulating hormone (FSH) was 11.5 mIU/mL, prolactin was 22 ng/mL, and anti-Müllerian hormone (AMH) was 3.5 ng/mL. These values provide an overview of the patient's endocrine function, reflecting normal ranges for the various hormones evaluated. Table 2 shows the hormonal profile report of the female patient. 

**Table 2:** Female hormonal profile. LH: luteinizing hormone; FSH: follicle-stimulating hormone; AMH: anti-müllerian hormone; mIU: milli international units; ng: nanogram; dL: deciliter; pg: picogram

Parameters	Reference values	Results
LH	2-10 mIU/mL	4.2 mIU/mL
Testosterone	15-70 ng/dL	22 ng/dL
Progesterone	0.1-0.7 ng/mL	0.4 ng/mL
Estradiol	30-400 pg/mL	110 pg/mL
FSH	4.7-21.5 mIU/mL	11.5 mIU/mL
Prolactin	<25 ng/mL	22 ng/mL
AMH	1.5-4.0 ng/mL	3.5 ng/mL

The female patient was advised to undergo a hormone test. The hormonal profile of the female patient revealed that all hormonal levels were within normal ranges. Based on the semen analysis report, the diagnosis was established. The semen analysis confirmed teratozoospermia, indicating that the male factor is the primary cause of infertility.

Therapeutic intervention

The IVF-ICSI cycle was recommended to the couple using the ZP sperm sorting technique. For the female patient, the gonadotropin-releasing hormone (GnRH) antagonist's short protocol was used to stimulate the ovaries on the third day of the menstrual cycle by combining with 5 milligrams (mg) of letrozole until the follicles reached 14-16 millimeters (mm) in diameter. Once the follicles reached an average diameter of 16 mm, 10,000 international units (IU) of human chorionic gonadotropin (hCG) were administered. After the ovum pick-up (OPU), seven oocytes were retrieved on day 12, including two in the metaphase I (MI) stage and five in the metaphase II (MII) stage.

**Figure 1:**
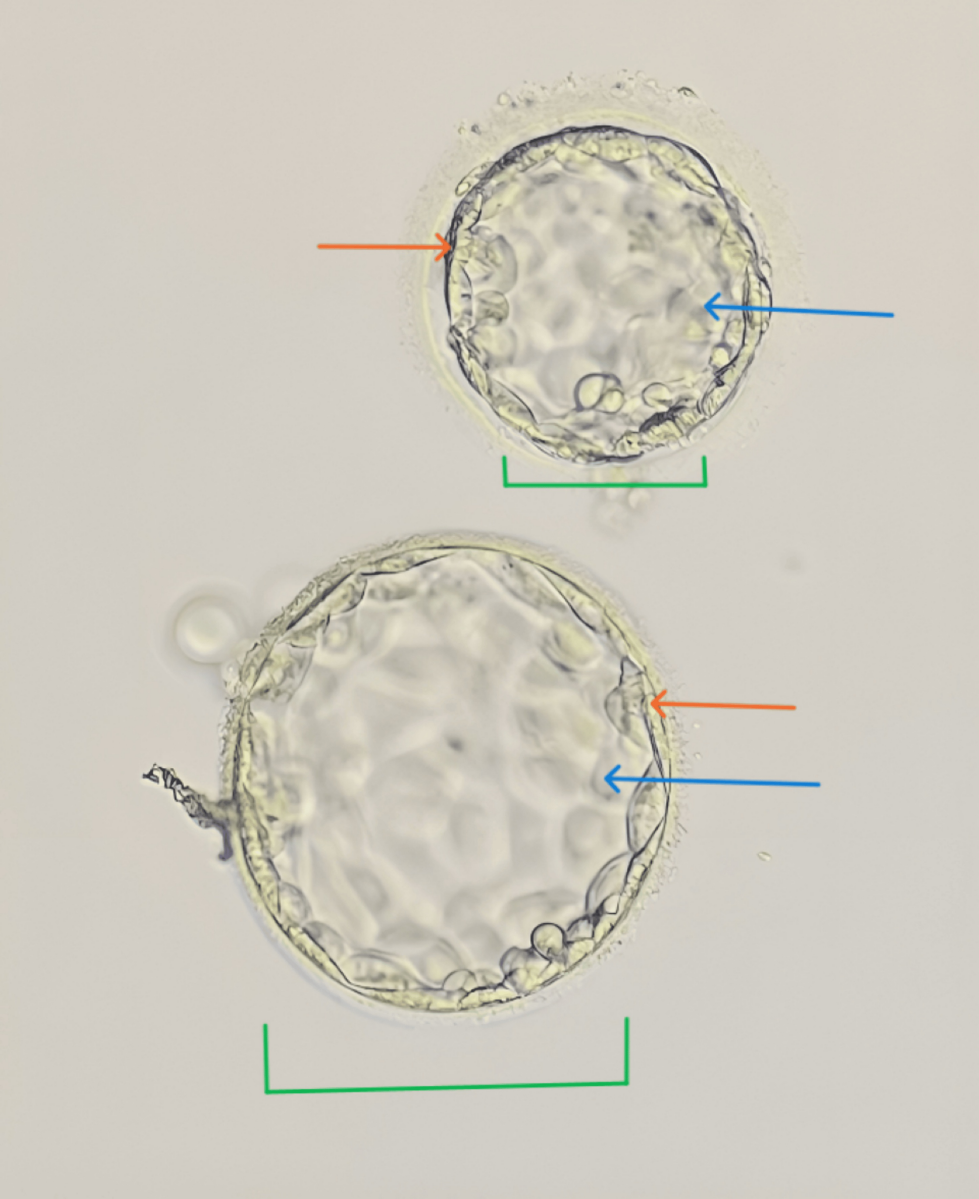
Grade 4BA and 3BB day five blastocyst The green bracket shows the expansion of the blastocyst; the orange arrow shows the inner cell mass (ICM); and the blue arrow shows the trophectoderm.

The ZP procedure was conducted following a specific protocol designed for sperm sample preparation. Since sperm cells lose negative electrical charge during capacitation, the immediate application of the zeta method was crucial. A new centrifuge tube was utilized, and electrostatic voltmeters were employed to measure the highest electrostatic charge. The process consisted of dispensing 0.1 mL of washed sperm into a tube and adding 5 mL of HEPES-human tubal fluid (HTF) medium. The tube was then rotated inside a latex glove to confirm a positive charge (~2 to ~4 kV at 1 inch). Tubes were kept at room temperature for one minute to allow sperm to adhere while preventing grounding. Glass centrifuge tubes were noted for better sperm binding than polystyrene. After centrifuging at 300 g for five minutes, non-adhering sperm and excess cells were removed by tube inversion and blotting. Adhering sperm on tube walls were rinsed with 0.2 mL of HEPES-HTF medium to eliminate the electric field, and the retrieved material was pipetted for subsequent sperm collection. Zeta method confirmation via an electrostatic voltmeter validated successful sperm selection. The ICSI procedures were performed on each mature oocyte, resulting in the fertilization of five oocytes. Subsequently, all five blastocysts were cryopreserved. After one month, during the initial round, two frozen embryos with grades 4BA and 3BB were chosen for embryo transfer (ET) (Figure 1). On day six of progesterone, ET was done with an endometrium thickness of 9 mm. The doctor instructed the patient to avoid physical exertion and schedule a follow-up appointment at the clinic in two weeks.

Follow up

Furthermore, the patient was prescribed progesterone, calcium, multivitamins, iron supplements, calciferol sachets for calcium intake, and prednisolone (5 mg). After two weeks, a urine pregnancy test (UPT) was done, which resulted in a positive, and the beta (β)-hCG level was 712 mIU/ml, indicating pregnancy. Sonography showed that the fetus was growing at a normal rate.

## Discussion

A large percentage of couples worldwide struggle with infertility [3]. Even though feminine factors are frequently given priority, male infertility plays a significant role in situations of ineffective conception [14]. This case study explains the difficulties faced by a couple struggling with infertility, primarily due to male-related issues, particularly abnormal sperm parameters and an elevated DFI. Although conventional ART methods like IVF-ICSI were initially utilized without significant success, the integration of individualized sperm selection approaches, such as ZP-based sorting, has demonstrated encouraging results. This is represented by the positive outcome of ET and the subsequent successful pregnancy observed.

Teratozoospermia arises from abnormal cellular differentiation during the process of spermatogenesis, and its etiology has been linked to various genetic and environmental influences, alongside factors such as advanced paternal age and psychological stress [10]. According to Brahem et al., infertile men with teratozoospermia showed a greater prevalence of chromosomal abnormalities and higher DFI as compared to normozoospermic men [15]. A high DFI, in this case, is an agreement with the paper stating the reason for the infertility faced by the couple was due to the male factor. Candela et al., in their paper, reported a correlation between abnormal sperm morphology and high blood pressure, which could also be a similar possibility [16].

Standard seminal preparation methods commonly utilized in ART include density gradient centrifugation (DGC) and swim-up procedures. These methodologies primarily rely on principles of sedimentation or migration to isolate optimal spermatozoa, particularly those exhibiting normal morphology and high nuclear maturity. However, these techniques do not directly assess or ensure the integrity of other critical sperm characteristics such as DNA integrity, apoptosis, membrane maturation, and ultrastructure. This limitation becomes more pronounced, particularly when starting with semen samples demonstrating asthenozoospermia and/or teratozoospermia. Consequently, these seminal preparation techniques do not guarantee the genomic integrity of the selected spermatozoa [17]. Therefore, ZP-based sperm selection is used as a novel approach to improve the efficacy of ART procedures in cases of male factor infertility.

The ZP method integrates DGC with selection based on the electric potential of the sperm membrane to isolate spermatozoa exhibiting a negative charge ranging from -16 mV to -22 mV. This negative charge range serves as evidence of adequate epididymal maturation and spermatogenesis occurring at the testicular level [18]. The selected spermatozoa demonstrate significant favorable alterations, including appropriate nuclear compaction, exchange of nuclear proteins, and accumulation of clusters of differentiation-52 (CD-52) glycopeptides enriched with sialic acid residues. Notably, the accumulation of sialic acid residues is a pivotal characteristic of the sperm maturation process, primarily responsible for the negative charge observed in the sperm plasma membrane [19]. Nasr Esfahani et al. demonstrated that the ZP method yields enhancements in the proportion of high-quality embryos and pregnancy rates compared to the DGC technique. This finding aligns with the outcomes observed in this case, where the implementation of the ZP method resulted in the generation of high-quality embryos, ultimately culminating in a successful pregnancy outcome [20].

## Conclusions

The use of zeta sperm selection in the treatment of male-factor infertility associated with excessive DNA fragmentation and ICSI results has been determined. A positive effect on fertility levels, embryo fertilization, and pregnancy has been seen when applying the ZP method for sperm separation. These outcomes confirm that the efficiency of the zeta-based approach can be one of the most beneficial alternatives for addressing the problem of sperm DNA fragmentation in ART outcomes.
